# Metabonomics Study of Essential Hypertension and Its Chinese Medicine Subtypes by Using Gas Chromatography-Mass Spectrometry and Nuclear Magnetic Resonance Spectroscopy

**DOI:** 10.1155/2013/625906

**Published:** 2013-02-25

**Authors:** Yunlun Li, Lei Nie, Haiqiang Jiang, Jiamao Lin, Honglei Zhou, Jun Xie, Zhengjun Qu, Dongmei Qi, Yunhui Zhang

**Affiliations:** ^1^Department of Cardiology, Affiliated Hospital of Shandong University of Traditional Chinese Medicine, Jinan 250011, China; ^2^School of Pharmaceutical Sciences, Shandong University, Jinan 250012, China; ^3^Experimental Centre, Shandong University of Traditional Chinese Medicine, Jinan 250355, China

## Abstract

A metabonomic study was performed to investigate the metabolic mechanism of essential hypertension and its Chinese medicine subtypes, including “Yin-deficiency and Yang-hyperactivity syndrome” (YDYHS) and “Yin-Yang deficiency syndrome” (YYDS). Plasma samples from 22 healthy volunteers, 31 hypertensive patients with YDYHS, and 29 hypertensive patients with YYDS were analyzed by ^1^H-NMR spectroscopy and gas chromatography coupled with mass spectrometry (GC-MS). The three groups were distinctly classified by principal components analysis (PCA) and partial least squares discriminant analysis (PLS-DA). According to identified biomarkers and their related pathways, abnormal glucose metabolism might be the main common pathway from YDYHS to YYDS, and sympathetic nervous system activation would play an important role in the pathogenesis of YDYHS, while a low metabolic rate usually occurred in YYDS.

## 1. Introduction

Essential hypertension (EH), a kind of genetic, heterogeneous complex disease, is very prevalent worldwide. Yet, the definite pathogenesis of EH is not clear. High blood pressure is merely a part of disease chain in metabolic disorder. Many metabolic factors are involved in the process of EH, which can increase the risk of damage of vascular endothelial cell and kidney [1]. The pathological process and characteristics of EH have been studied by modern medicine and traditional Chinese medicine (TCM) from different viewpoints for many years. In addition to modern medicine generally using chemical drugs for EH treatment, TCM is widely employed as a quite important therapeutic strategy by using acupuncture or TCM herbal formulae in current Chinese medicine clinical practice. Yet, these two medical systems gain insight into EH from very different perspectives. The treatment goal of Western medicine aims at changes in blood pressure and has a great superiority in the local characterization of EH, whereas TCM cares more about the pathological changes of EH patients and mainly focuses on physiological changes from a holistic perspective [2]. The overall information about patient's symptoms and signs judged by the Eastern practitioners is the main basis of Chinese medicine diagnosis. And according to TCM theory, all the related symptoms and signs in a certain disease phase are generalized to a syndrome (“Zheng” in Chinese medicine), which is the basic unit and a key concept of TCM [3]. Thus, patients with the same disease can be divided into different syndromes (e.g., different Zhengs). According to the theory of “Zheng” in TCM, the basic nature of Yin and Yang is that Yin and Yang are seemingly two contrary forces and can be balanced and transformed into each other [4]. In the diagnosis of EH, the “Yin-deficiency and Yang-hyperactivity syndrome” (YDYHS) and the “Yin-Yang deficiency syndrome” (YYDS) are the two main subtypes diagnosed from the viewpoint of TCM and about 10 clinical practice guidelines [5]. When Yin is insufficient, Yang loses its restraint stemmed from Yin and becomes relatively predominant, and then YDYHS will accordingly happen. The patients with YDYHS often show some symptoms such as headache, dizziness, tinnitus, irritability, hot face, and weak waist. If YDYHS lasts for a long time, the capability of mutual transformation between Yin and Yang will be reduced, and Yang will become also deficient. Subsequently, the Yin-Yang deficiency syndrome (YYDS) will occur. People suffering from YYDS still manifest the syndromes such as headache, dizziness, fatigue, easily catching cold, spontaneous perspiration, and palpitation [6]. However, the description for differentiating the two types of syndrome in TCM is rather abstract, so it is very essential to develop new method to give a more objective representation.

Metabonomics, a new omics technique concerning the global information of metabolites in living systems and their dynamic responses to either endogenous changes, exogenous stimuli, or genetic manipulation [7], has widely demonstrated its potentials to explore the biological mechanisms of “Zheng” in TCM. There are specific metabolism patterns in different physiological and pathological stages, and the alteration of metabolism is closely correlated with the level of physiology and pathology in entrails [8]. Therefore, the connotation of “Zheng” in TCM could be better revealed based on metabonomics, and the dynamic feature of “Zheng” could be expressed as well [9]. In the present paper, metabonomics method was employed to investigate the essence of YDYHS and YYDS in EH. Several analytical techniques, including ^1^H-NMR and mass spectrometry (MS), have been widely used in the field of metabonomics. NMR is an early technique used in metabonomics. Whereas, ^1^H-NMR analysis is restricted to a limited number of high-concentration metabolites. An alternative approach is liquid chromatography (LC) or gas chromatography (GC) combined with mass spectrometry (MS), which can offer higher sensitivity compared to ^1^H-NMR. Thus, GC-MS or LC-MS not only can be used to detect low-concentration metabolites but also can be employed to identify the structure of biomarkers. Nevertheless, it should be necessary to recognize that ^1^H-NMR spectroscopy and mass spectrometry are complementary tools, and the combination will provide more detailed information in metabonomics studies [10–12].

Recently, metabonomic approach has been applied to the research of “Zheng” types of hypertension in modern TCM. GC/MS-based metabonomics method was used to investigate plasma metabolomic mechanism of healthy volunteers and hypertensive patients who were divided into three types of “Zheng,” including “liver-fire flaming syndrome,” “phlegm flourishing syndrome,” and YDYHS. The results showed that normal plasma samples could be distinguished from those of the three TCM “Zheng” types, and the metabonomic profiles of plasma between “Liver-fire Flaming Syndrome” and YDYHS were markedly different [13].

Metabonomics offered a fresh insight into relations and differences among different types of “Zheng”. In this paper, plasma metabonomics study of EH and its two Chinese medicine subtypes was carried out by using ^1^HNMR and GC-MS. Firstly, the plasma metabolic characteristics of EH and its two subtypes were studied to gain a deep understanding of the metabolic perturbations associated with disease and to explore the metabolite evidence in TCM theory. Secondly, the related metabolism network was constructed according to biochemical reactions associated with identified biomarkers. Finally, the metabolic tendency of biomarkers in different groups was also analyzed to explore the possible metabolic mechanism of YDYHS and YYDS.

## 2. Materials and Methods

### 2.1. Chemicals and Reagents

Deuterium oxide 99.9% (D_2_O) containing 0.05% trimethylsilyl-2,2,3,3-d_4_ propionic acid sodium salt (TSP), pyridine 99.8%, methoxyamine hydrochloride 98%, and N-methyl-N-(trimethylsilyl)trifluoroacetamide (MSTFA) were purchased from Sigma-Aldrich Ltd. (Oakville, ON, Canada). Methanol (99.9%) was purchased from Fisher Scientific Company (Ottawa, ON, Canada).

### 2.2. Sample Collection and Preparation

This study was reviewed and approved by the Ethics Committee of Shandong University of Traditional Chinese Medicine. The institutional review board approved the protocol, and all patients provided a written informed consent. Patients (age range: 18–60 years of age) were eligible if they met the diagnostic criteria of YDYHS and YYDS, and their diastolic blood pressure was between 90 mmHg and 109 mmHg after a two-week washout period. Patients with diabetic mellitus, uncontrolled hypertension, heart failure, renal dysfunction, or liver disease were excluded. Samples collected from all patients matching the inclusion criteria and healthy volunteers were analyzed by ^1^H-NMR or GC-MS.

Blood samples were collected from vein in the morning with anticoagulation and stored in −80°C refrigerator for further analysis. Prior to analysis, plasma samples were thawed at 4°C. Two hundred and fifty microlitres of methanol was added into 100 *μ*L of plasma for protein precipitation (kept on ice for 15 min) and immediately centrifuged at 11,200 g for 10 min. Afterwards, the supernatant was pipetted out and lyophilized.

### 2.3. NMR Analysis

The lyophilized samples were dissolved by 600 *μ*L D_2_O mixed with TSP (0.05% (w/v) in D_2_O) as an internal chemical shift reference (**δ**0). The sample supernatants were then transferred into 5-mm NMR tubes.

A Bruker superconducting NMR spectrometer was utilized to detect the samples with the pulse sequence “cpmgpr1d” to eliminate the interference of macromolecular compound in blood. A total of 64 transients and 32000 data points were collected. The following parameters were set for NMR detection: spectral width: 7288.63 Hz; recycle delay (RD): 2 s; and acquisition time: 2.6 s. Water suppression was adopted by using low-power presaturation during RD and mixing period (*t*_*m*_ = 0.15 s). Before Fourier transformation (FT), the free induction decay (FID) signals were zero filled by a factor of 2 and multiplied by an exponential line-broadening function of 0.5 Hz. The phase correction, baseline correction, and chemical shift reference to TSP (**δ**0) were carried out manually for preprocessing the available spectra.

### 2.4. GC-MS Analysis

Fifty microlitres of methoxyamine hydrochloride (20 mg/mL pyridine) was used to dissolve dried residue, which stayed at 20°C for 24 h. Then 75 *μ*L of MSTFA (with 1% TMCS) was added to interchange the acidic protons at 37°C for 1 h. In the end, one hundred and fifty microlitres of n-heptane was employed to stop the reaction.

Agilent 6890N gas chromatography and a quadruple mass spectrometer equipped with a HP-5 MS capillary column (30 m × 0.25 mm i.d. 0.32 *μ*m thickness) were used to analyze the supernatant in vial. The injector temperature was 270°C. A constant flow rate of 1 mL/min of helium carrier gas was set. The initial temperature of GC oven was held at 85°C for 5 min and then ramped to a final temperature of 300°C at a rate of 10°C/min with a 5 min hold time. The temperatures of transfer line, ion source, and quadrupoles were set at 270°C, 230°C, and 150°C, respectively. Ionization was achieved by a 70 eV electron beam at a current of 2.0 mA. The solvent delay time was set at 2 min. Mass spectra were obtained with a mass-to-charge ratio (*m*/*z*) of 50–550 [14]. 

### 2.5. Data Analysis

Two hundred and twenty five integral regions of equal width (0.04 ppm) were obtained from the NMR spectra data of plasma samples in a range of **δ**10–**δ**0 using MestRe-C (version 4.9.9.9, http://www.mestrec.com/). The region within **δ**4.5–**δ**5.0 was deleted to eliminate the chemical shift of water. After GC-MS analysis, seventy-five common peaks and their peak areas were acquired from the total ion chromatograms (TICs) according to the retention time. The common characteristic peaks were saved as excel files for further data analysis.

Principal component analysis (PCA) and partial least-squares discriminant analysis (PLS-DA) were implemented by SIMCA-P (version 11.5, Umetrics, Umea, Sweden) for NMR and GC-MS data analysis. Before PCA and PLS-DA, all variables were preprocessed by normalization to clear the influence of uncorrelated factors such as inconstancy in the process of sample preparation. The *R* (the multiple correlation coefficient) and the *Q* (the cross-validated correlation coefficient), which revealed the fraction of the total variation predicted by components, were used to measure the robustness of a pattern recognition model [15]. The two values of *R* and *Q* could be used to evaluate model fit and predictive ability, respectively. Variable influence on projection (VIP) provided by SIMCA-P program was employed to compare the relative influence of variables according to their contribution to the model.

### 2.6. Structure Identification of Potential Biomarkers

The potential biomarkers were selected according to the VIP values greater than 1, which was generally used as a criterion for variable selection [16]. For the data of NMR spectroscopy, the chemical shift of compound was inquired for identification in the database including METLIN, HMDB, and KEGG. For GC-MS data, the *m*/*z* value was searched in NIST2008 database.

## 3. Results and Discussions

### 3.1. Sample Characteristics

A total of 11 healthy volunteers, 14 hypertensive patients with YDYHS, and 10 hypertensive patients with YYDS were allocated to NMR analysis, while 11 healthy volunteers, 17 hypertensive patients with YDYHS, and 19 hypertensive patients with YYDS were allocated to GC-MS analysis. Demographic characteristics were shown in Tables 1 and 2. Seen from Tables 1 and 2, for both NMR and GC-MS analysis, there were no significant differences among the three groups in age and sex.

### 3.2. Analysis of Plasma Samples by NMR Spectroscopy and GC-MS

NMR spectroscopy and GC-MS were used to acquire the metabolic profile of plasma samples from hypertension patients with YDYHS, hypertension patients with YYDS, and healthy volunteers. 

**Figure 1:**
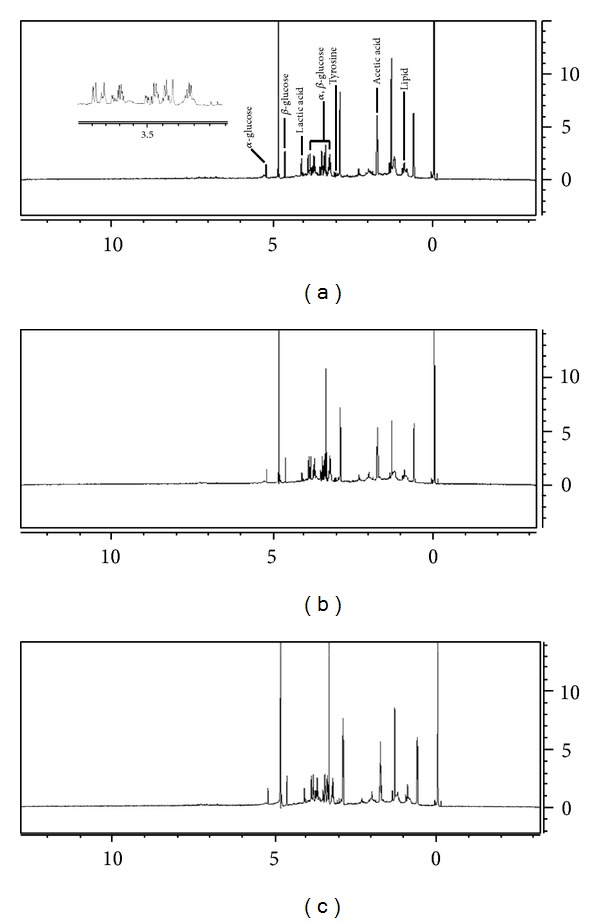
Representative ^1^H-NMR spectra of plasma samples from (a) hypertension patients with YDYHS, (b) hypertension patients with YYDS (c), and healthy volunteers.

**Figure 2:**
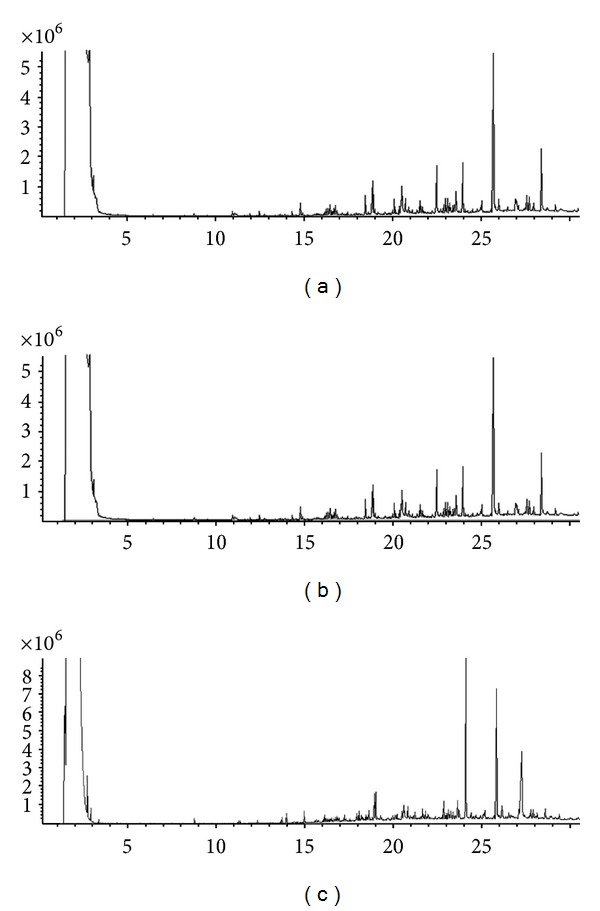
Representative GC-MS TICs of plasma samples from (a) hypertension patients with YDYHS, (b) hypertension patients with YYDS (c), and healthy volunteers.

The representative NMR spectra from different groups were shown in Figure 1. The main components of plasma are glucose, organic acids, amino acids, and lipid compounds. The typical total ion chromatograms of GC-MS of plasma samples were displayed in Figure 2. Seventy-five common peaks were selected from TICs of GC-MS as the endogenous metabolites, including amino acids, fatty acids, sugars, and organic acids.

### 3.3. Data Analysis

**Figure 3:**
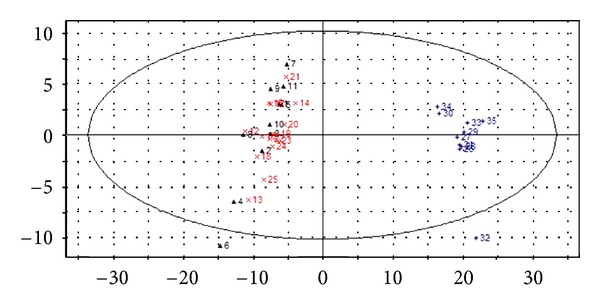
PCA score scatter plot of plasma samples from NMR data of hypertension patients with YDYHS (Number 12–25), hypertension patients with YYDS (Number 26–35), and healthy volunteers (Number 1–11).

**Figure 4:**
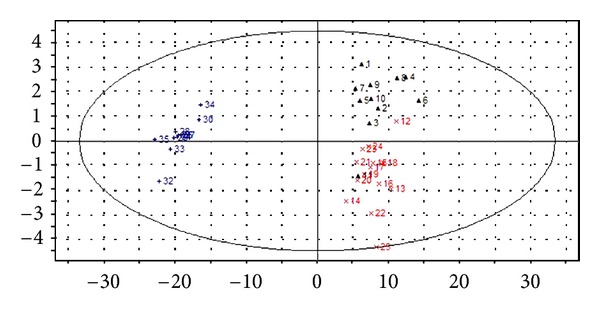
PLS-DA score scatter plot of plasma samples from NMR data of hypertension patients with YDYHS (Number 12–25), hypertension patients with YYDS (Number 26–35), and healthy volunteers (Number 1–11).

**Figure 5:**
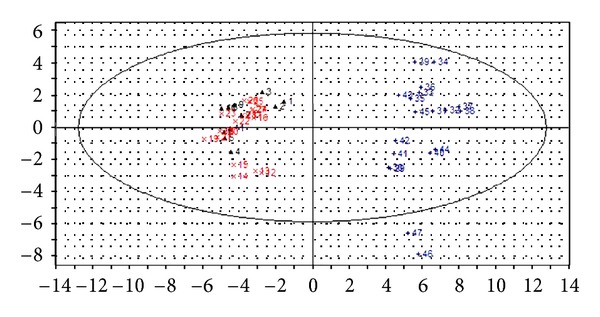
PCA score scatter plot of plasma samples from GC-MS data of hypertension patients with YDYHS (Number 12–28), hypertension patients with YYDS (Number 29–47), and healthy volunteers (Number 1–11).

**Figure 6:**
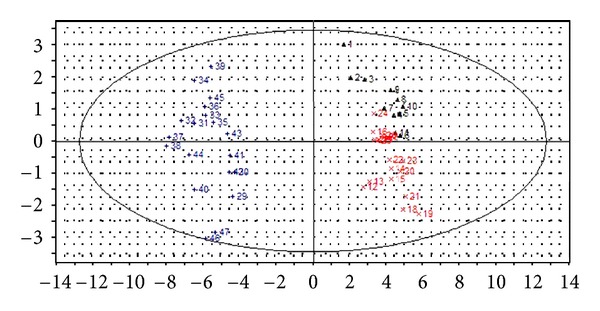
PLS-DA score scatter plot of plasma samples from GC-MS data of hypertension patients with YDYHS (Number 12–28), hypertension patients with YYDS (Number 29–47), and healthy volunteers (Number 1–11).

**Figure 7:**
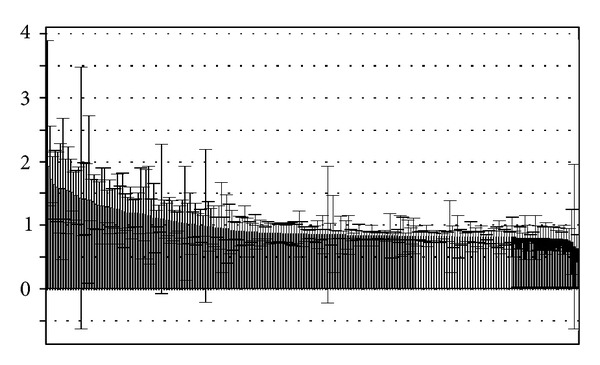
VIP plot of PLS-DA based on NMR data.

**Figure 8:**
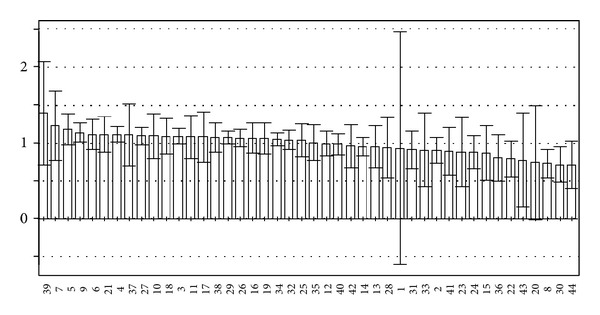
VIP plot of PLS-DA based on GC-MS data.

The PCA score scatter plots were calculated based on the subsection integral values of NMR spectra and the peak areas of GC-MS TICs (shown in Figures 3 and 5), respectively. There is no overlap between YDYHS and YYDS samples, suggesting the two groups are obviously different in metabolism mode. Since YDYHS patients and healthy volunteers are mixed together (see Figures 3 and 5), PLS-DA was used for further data analysis. The classification ability of PLS-DA is markedly improved compared with PCA for NMR and GC-MS data (shown in Figures 4 and 6). For NMR data, the value of *R* ^2^ is 0.878, which indicated that the first five PLS components can explain 87.8% of total variance of *Y* (i.e., category information). The prediction ability of the model reaches 71.4% (*Q* ^2^ = 0.714). For GC-MS data, the value of *R* ^2^ is 0.770, which denotes that the first three PLS components can explain 77.0% of total *Y* variance. The prediction ability of the model is 65.1% denoted by *Q* ^2^ (0.651). The values of *R* ^2^ and *Q* ^2^ suggest that the models based on NMR and GC-MS data both have a strong fitting ability and predictive ability. The variables of VIP values (see Figures 7 and 8) greater than 1 given by PLS-DA were selected because they were considered to be influential for classification [16]. The compounds corresponding to these significant variables were regarded as the potential biomarkers. 

Through PCA and PLS-DA methods, three groups were successfully classified. The results based on NMR and GC-MS data are similar. According to the results, YDYHS group is partly mixed with healthy volunteers, but YYDS group does not overlap with the other two groups, indicating that YDYHS and YYDS might belong to two different metabolic categories of hypertension. The metabolic mode of YDYHS patients compared with that of YYDS patients is closer to healthy volunteers. Therefore, the result shown in PCA score plot is consistent with TCM theory, which is that YDYHS happens in early EH while YYDS appears in further stage [17].

### 3.4. Biomarkers and Metabolic Perturbations

According to the VIP plots of PLS-DA (shown in Figures 7 and 8), the corresponding chemical shifts and *m*/*z* values of significant variables contributed to classification were found. The databases including HMDB, KEGG, METLIN, LIPID MAPS, and PUBCHEM [18] were used to retrieve the structures of compounds according to their chemical shifts. A NIST2008 database was employed to identify the structures of compounds based on *m*/*z* values. The related metabolic pathways were searched in KEGG database. The changed metabolic patterns induced by hypertension resulted in the changed metabolic trends. The identified biomarkers and their relative changes were shown in Table 3.

TCM considers that Yin deficiency is the common pathological change for YDYHS and YYDS. Therefore, if any metabolite showed the same metabolic tendency in two “Zhengs”, it could be regarded as the biomarker of Yin deficiency of hypertension. In Table 3, compared to control group, glucose showed an increasing tendency in both of the YDYHS and YYDS groups. Therefore, it suggests that glucose may be a metabolic biomarker of Yin deficiency in hypertension, and abnormal metabolism of glucose may be a metabolic pathway of Yin deficiency in hypertension. 

In hypertension, disorders of glucose are usually caused by insulin resistance [19]. The recent experiments showed that insulin resistance played an important role in increasing the risk of EH [20, 21]. Both sympathetic nerve system activity [22, 23] and activating renin-angiotensin-aldosterone system caused by insulin resistance play critical roles in the pathological mechanism of EH [24, 25]. Furthermore, the previous experiments gave results that hypertensive patients with YDYHS and YYDS had the same symptom of insulin resistance compared to healthy people, and YYDS was more serious in insulin resistance than YDYHS [26, 27]. 

### 3.5. Metabolic Pathway Analysis

**Figure 9:**
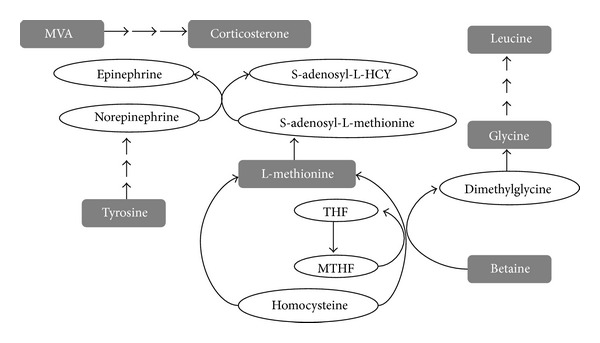
Metabolic network of Yang hyperactivity in hypertension. The compounds in gray rectangle refer to the identified biomarkers.

The biomarkers and their metabolic pathways merely show the isolated change of EH. However, according to biochemical reaction, the metabolic network built by combining the biomarkers with their corresponding metabolic pathways can display the holistic changes in human body (see Figure 9).

The varying levels of biomarkers (see Table 3) in YDYHS seem to be a reasonable consequence of the sympathetic nervous system activation, which are the key factors in the etiology of EH [28]. As shown in Figure 9, tyrosine is a precursor for the synthesis of norepinephrine and epinephrine, while betaine and L-methionine can supply methyl for norepinephrine and epinephrine. Mevalonic acid, an important semifinished product in the process of cholesterol synthesis, is generated by acetyl-CoA with the catalysis of HMG-CoA, which can be activated by epinephrine. Cholesterol can be transformed to adrenocortical hormone, such as corticosterone [29]. Consequently, tyrosine, betaine, L-methionine, and mevalonic acid (MVA) are required in more quantities during the process of sympathetic nervous system activation, which might be an important mechanism of pathogenesis of YDYHS in EH. 

Yang, as a TCM conception, refers to a cluster of material resources including warmth, excitement, and promotion [4], and the sympathetic nervous system activation is an important pathological progress of Yang hyperactivity [30]. The results of previous experiments were similar to the present study that the levels of norepinephrine and epinephrine in the blood of hypertensive patients with YDYHS were higher than that of healthy people and YYDS patients. Therefore, the levels of norepinephrine and epinephrine might be thought to be the important diagnostic criteria for YDYHS of EH.

**Figure 10:**
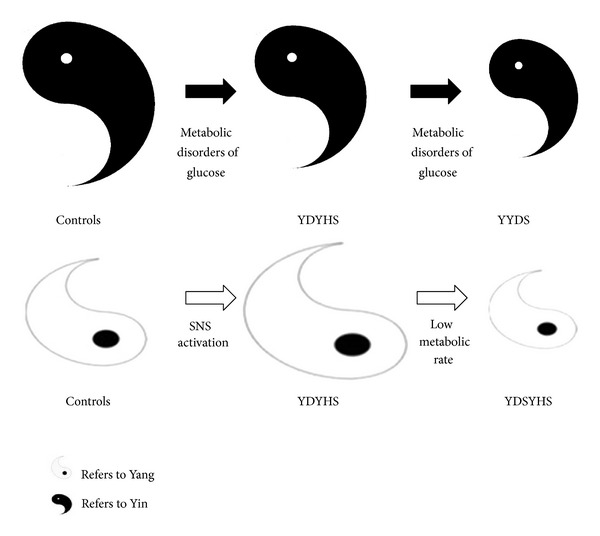
Metabolic mechanism in EH based on the relative changes of Yin and Yang.

Further, according to TCM theory, Yang can also keep metabolic rate in a normal level to satisfy the normal physiological activities of the human body [31]. However, for YYDS of EH, the normal physiological function is depressed and the body is usually in a low metabolic status. The patients with YYDS always exhibit a series of Yang-hypofunction symptoms such as cold, weakness, poor appetite, and listlessness. As illustrated in Table 3, comparing to YDYHS and control group, all biomarkers except for glucose showed a decreased metabolic tendency in YYDS, and they might be regarded as the characteristic biomarkers of Yang hypofunction. As shown in Figure 10, metabolic disorders of glucose may be the shared pathway of YDYHS and YYDS. However, different metabolic modes are generated as the result of alteration in the two “Zhengs”. 

## 4. Conclusion

The objectification of “Zheng” in TCM theory is a very difficult issue in the modern medical research. The incorporation of TCM pattern classification and biomedical disease diagnosis will lead to a new era with the development of medical sciences. Improving treatment effectiveness and more methods applied to diagnosis, clinical trial, and new drug discovery in TCM will be provided [32, 33]. Metabonomics was demonstrated as a useful tool for understanding this kind of incorporation. However, different metabolic modes were generated due to alteration in two different “Zhengs”. The sympathetic nervous system activation may be an important pathogenesis in YDYHS characterized by Yang hyperactivity, while lowered metabolic rate usually occurred in YYDS. It was also worth noticing that the complementary nature of the data obtained by GC-MS and NMR was illustrated in the results of the present study, so more metabolic biomarkers could be identified than those obtained by any single technique [34]. Hence, the combination of different metabonomics techniques would help us to discover and reveal the metabolic modes in different “Zheng” of TCM.

## Figures and Tables

**Table 1:** Basic demographic characteristics of participants in NMR analysis.

Characteristics	Controls% (*n*)	YDYHS group% (*n*)	YYDS group% (*n*)	*P* value
Sex				1.000
Female	45.45 (5)	42.86 (6)	40 (4)	
Male	54.56 (6)	57.14 (8)	60 (6)	
Age				1.000
30–40	9.09 (1)	14.29 (2)	10 (1)	
40–50	36.36 (4)	35.71 (5)	40 (4)	
50–60	54.55 (6)	50 (7)	50 (5)	

**Table 2:** Basic demographic characteristics of participants in GC-MS analysis.

Characteristics	Controls% (*n*)	YDYHS group% (*n*)	YYDS group% (*n*)	*P* value
Sex				0.913
Female	54.55 (6)	47.06 (8)	47.37 (9)	
Male	45.55 (5)	52.94 (9)	52.63 (10)	
Age				0.941
30–40	9.09 (1)	5.88 (1)	10.53 (2)	
40–50	36.36 (4)	52.94 (9)	42.11 (8)	
50–60	54.55 (6)	41.18 (7)	47.37 (9)	

**Table 3:** Potential biomarkers, metabolic pathways, and metabolic tendencies of biomarkers from YDYHS to YYDS.

Number	Instrument	Metabolites	Metabolic pathway	Metabolic tendency		
				YDYHS versus controls	YYDS versus controls	YYDS versus YDYHS
1	NMR	Betaine	Epinephrine biosynthesis	↑	↓	↓
2	NMR	Mevalonic acid	Cholesterol biosynthesis	↑	↓	↓
3	NMR	Corticosterone	Steroid hormone biosynthesis	↑	↓	↓
4	NMR	Beta-leucine	Valine, leucine, and isoleucine degradation	↑	↓	↓
5	NMR	Propionic acid	Cysteine and methionine metabolism	—	↓	↓
6	GC-MS	Methionine	Cysteine and methionine metabolism	↓	↓	↓
7	GC-MS	D-glucose	Tricarboxylic acid cycle	↑	↑	↓
8	GC-MS	Glycine	Glycine, serine, and threonine metabolism	↑	↓	↓
9	GC-MS	Tyrosine	Tyrosine metabolism	↑	—	↓
10	GC-MS	Malic acid	Fatty acid biosynthesis	↑	↓	↓
